# Predicting cognitive decline with non-clinical markers in Parkinson’s disease (PRECODE-2)

**DOI:** 10.1007/s00415-019-09250-y

**Published:** 2019-02-28

**Authors:** Tayyabah Yousaf, Gennaro Pagano, Flavia Niccolini, Marios Politis

**Affiliations:** Neurodegeneration Imaging Group, Institute of Psychiatry, Psychology and Neuroscience (IoPPN), Maurice Wohl Clinical Neuroscience Institute, King’s College London, 125 Coldharbour Lane, Camberwell, London, SE5 9NU UK

**Keywords:** Parkinson’s disease, Cognitive decline, Cognitive impairment, Predictors, CSF, [^123^I]FP-CIT SPECT

## Abstract

**Objectives:**

To investigate whether baseline [^123^I]FP-CIT SPECT and CSF markers can predict cognitive impairment (CI) in PD patients, and provide a profile of those most at risk.

**Methods:**

262 de novo PD patients from the Parkinson’s Progression Markers Initiative database were stratified into two CI groups at the 36-month follow-up: MoCA-defined diagnosis: PD patients who had a MoCA score < 26; neuropsychological test-defined diagnosis: PD patients with MoCA-defined diagnosis and at least two test scores (of six; irrespective of test domain) greater than 1.5 standard deviation below the mean score in healthy controls. Predictive variables of CI were divided into deciles, providing us with ideal cutoff values for each variable.

**Results:**

At the 36-month follow-up, 108/262 (41.2%) PD patients had CI as defined by the MoCA, of which 40/108 (37.0%) had neuropsychological test-defined CI. Baseline CSF Aβ42 (hazard ratio [HR]: 0.996, confidence interval [CI]: 0.992–0.999, *p* = 0.025), CSF total tau ([HR]: 1.023, [CI]: 1.002–1.044, *p* = 0.031) and caudate [^123^I]FP-CIT SPECT uptake ([HR]: 0.332, [CI]: 0.115–0.960, *p* = 0.042) were predictors of CI. Patients with reduced CSF Aβ42 (< 384.6 pg/mL), increased CSF total tau (> 45.0 pg/mL) and reduced caudate [^123^I]FP-CIT SPECT uptake (< 1.82) had a 65% risk of developing CI at 36-month follow-up.

**Conclusion:**

We report a characteristic profile (reduced CSF Aβ42, increased CSF total tau and reduced caudate [^123^I]FP-CIT SPECT uptake) that enables identification of early PD patients at risk of developing CI. These findings confirm previous reports of low CSF Aβ42, elevated CSF total tau and reduced dopaminergic integrity being associated with cognitive decline in PD.

## Introduction

Cognitive impairment (CI) is an increasingly recognised complication of Parkinson’s disease (PD), with significant impact including increased caregiver burden and mortality [1]. Mild cognitive impairment (MCI), in particular, is recognised as a distinct entity, and a probable prodromal state to PD dementia [1]. Up to 80% of Parkinson’s disease patients develop dementia during the course of the disease, presenting with heterogeneous cognitive profiles and underlying disease pathology [1]. It is crucial to identify CI at an early stage to determine cognitive prognosis, given that MCI is a key risk factor for dementia [15].

Discovering meaningful end points that measure progression is one of the greatest challenges in PD therapeutics. The implementation of suboptimal methods in refining prognosis, diagnosis and tracking disease progression continues to hamper patient management and, particularly, clinical research [27]. Thus, validated biomarkers with a high degree of sensitivity and specificity represent an unmet need in PD, especially for the development of disease-modifying therapies.

Previous reports have demonstrated that vascular and Alzheimer-type (amyloid-β plaques and tau neurofibrillary tangles) pathologies co-exist in PD, and may contribute to the development of CI in PD [16]. In PD, the degenerative process commences years before any initial signs of CI, and cerebrospinal fluid (CSF) markers could identify these pathological changes. This is because the CSF has more physical contact with the brain compared to other fluids, as it is not separated from the brain by the tightly regulated blood–brain barrier. As a result, proteins or peptides that may be directly reflective of disease pathology or brain-specific activities are likely to diffuse into the CSF, therefore highlighting that the CSF is potentially the most informative fluid in biomarkers discovery for neurodegenerative disease prognosis [5]. Previous studies have reported that reduced amyloid-β42 (Aβ42) [28] and increased total-tau and phosphorylated-tau [8] levels in CSF are associated with cognitive decline in PD.

Several PET studies have also reported that dysfunction of the dopaminergic system also influences cognition in PD, with frontostriatal dopaminergic deficits affecting executive function and cholinergic deficits affecting memory and visuospatial function [19]. Striatal dopaminergic function has also been implicated in cognitive performance in PD, with Jokinen and colleagues reporting a positive correlation between caudate [^18^F]DOPA uptake and performance in visual and verbal memory [17] and Rinnie and colleagues reporting an association between reduced caudate [^18^F]DOPA uptake and impairment in verbal fluency, working memory and attentional functioning [25].

These observations prompted the present study, which sought to determine whether the combined evaluation of different non-clinical markers would be more useful to identify PD patients most at risk of developing CI, as identifying a combination of imaging and CSF biomarkers of cognitive decline provides a fruitful and more accurate approach for diagnosis and disease progression, compared with using either modality alone.

In this study, we hypothesised that molecular markers of PD pathology may have value for predicting cognitive decline and we sought to investigate this by using longitudinal clinical data from the Parkinson’s Progression Markers Initiative (PPMI) and baseline [^123^I]FP-CIT single photon emission computed tomography (SPECT) imaging and CSF molecular measures in a group of patients, who at baseline, had early de novo PD.

## Materials and methods

### Participants and clinical characteristics

Data were acquired from the PPMI database (http://www.ppmi-info.org/data), which is a 5-year observational, international, multi-centre prospective study that provides an insight into disease aetiology by collecting biannual neurological and cognitive evaluations and baseline CSF sampling [22]. Of a total of 412 PD patients, we identified and included 262 PD patients who were not cognitively impaired at baseline (MoCA ≥ 26), had [^123^I]FP-CIT SPECT and CSF data, and a complete 36-month follow-up.

All PD patients underwent an initial screening visit followed by a baseline visit where demographic, CSF and [^123^I]FP-CIT SPECT measures were collected (Table 1). Motor, non-motor, cognitive and neuropsychological assessments were also collected at baseline. The follow-up period was either terminated at the time point when patients had developed CI (*n* = 108) or at the complete 36-month follow-up visit if they had not developed CI (*n* = 154).

**Table 1:** Demographic characteristics and CSF, plasma and imaging measures for PD patients who developed MoCA-defined cognitive impairment at the 3-year follow-up

	All PD patients (*n* = 262)	Cognitive impairment (*n* = 108)	No cognitive impairment (*n* = 154)
Age at screening, mean ± SD^a^	61.08 ± 9.47	64.56 ± 8.46	58.64 ± 9.41*
Gender, male, % (*n*)^b^	63.7 (167)	73.1 (79)	57.1 (88)*
Age at diagnosis, mean ± SD^a^	60.52 ± 9.45	63.97 ± 8.54	58.10 ± 9.33*
PD duration (months), mean ± SD^b^	6.68 ± 6.87	7.06 ± 7.11	6.40 ± 6.70
Family history of PD, % (*n*)^b^	25.6 (67)	24.1 (26)	26.6 (41)
Year of education, mean ± SD^b^	15.73 ± 2.97	15.61 ± 3.42	15.81 ± 2.62
CSF Aβ1–42 levels pg/mL, mean ± SD^b^	369.89 ± 93.38	350.04 ± 90.07	383.81 ± 93.43*
CSF alpha-synuclein pg/mL, mean ± SD^b^	1860.22 ± 774.80	1808.73 ± 715.97	1896.33 ± 813.87
CSF phosphorylated tau-181 pg/mL, mean ± SD^b^	15.53 ± 10.37	15.06 ± 11.33	15.87 ± 9.66
CSF total tau pg/mL, mean ± SD^b^	44.06 ± 17.14	45.58 ± 19.26	42.99 ± 15.45
Apolipoprotein A1 mg/dL, mean ± SD^b^	166.98 ± 34.87	167.11 ± 34.38	166.89 ± 35.51
Amyloid precursor protein, mean ± SD^b^	43.50 ± 2.64	43.68 ± 2.43	43.37 ± 2.82
Glucose mmol/L, mean ± SD^b^	5.48 ± 0.78	5.54 ± 0.85	5.44 ± 0.73
Serum IGF-1 ng/mL, mean ± SD^b^	138.92 ± 54.08	137.24 ± 56.29	140.10 ± 52.63
Uric acid umol/L, mean ± SD^b^	316.26 ± 77.52	334.41 ± 78.78	303.53 ± 74.26*
Caudate [^123^I]FP-CIT, mean ± SD^a^	1.96 ± 0.54	1.92 ± 0.59	1.99 ± 0.49
Putamen [^123^I]FP-CIT, mean ± SD^b^	0.82 ± 0.27	0.82 ± 0.31	0.83 ± 0.25

*p values < 0.05 (^a^*t* test,^b^Mann–Whitney *U* test)

Institutional review boards approved the study and written informed consent was obtained from all participants. All PD patients were recruited between 2010 and 2015, had a disease duration of less than 2 years prior to a screening visit, were not taking dopamine replacement therapy and presented with at least two of the following symptoms: bradykinesia, rigidity, and resting tremor, OR the presence of either an asymmetric resting tremor or asymmetric bradykinesia. PD diagnosis was confirmed by presence of dopamine transporter deficit on the [^123^I]FP-CIT dopamine transporter imaging.

### [^123^I]FP-CIT SPECT molecular imaging

SPECT images were obtained 4 ± 0.5 h after administrating an injection of approximately 185 MBq [^123^I]FP-CIT. [^123^I]FP-CIT SPECT scans were analysed following the imaging technical operations manual (http://ppmi-info.org/). In brief, SPECT image volumes were spatially normalised to an Ioflupane template. The eight most prominent axial slices containing the striatum were summed and a standardised volume of interest (VOI) template was then applied to this image. VOI analyses were performed on the right and left caudate and putamen, employing the occipital region as the reference tissue. Specific binding ratios were calculated as the ratio of the putamen or caudate VOI count density divided by the occipital cortex count density minus one. This measure approximates the binding potential, BP_ND_, when the radioligand is in equilibrium at the target site and has previously been reported with Ioflupane SPECT.

### CSF and plasma molecular markers

CSF and plasma samples were collected at baseline from all participants enrolled in the study including CSF Aβ42, CSF alpha-synuclein, CSF total-tau, CSF phosphorylated tau-181, serum insulin-like growth factor 1 (IGF-1), amyloid precursor protein (APP), apolipoprotein A1, EGF ELISA, urate and glucose. Additional information on how CSF samples were collected and analysed has been previously reportedly [18].

### Primary end point

Cognitive decline was the primary outcome of the study, which was reviewed and established by the study physicians during follow-up visits. The follow-up period was either terminated at the time-point when patients had developed CI or at the end of the study period if they had not developed CI. Individuals were classified as having normal cognition or cognitive impairment as outlined in the PPMI protocol. Cognitive decline was evaluated using the Montreal Cognitive Assessment (MoCA), which is a scale for assessing global cognitive abilities. Additionally, a detailed cognitive battery, as previously described [31], was administered: the Hopkins Verbal Learning Test-Revised for memory; the Benton Judgement of Line Orientation 15-item (split-half) version for visuospatial function; the Letter–Number Sequencing for executive function and working memory; the Symbol–Digit Modalities Test for processing speed–attention; and semantic (animals, fruits and vegetables) fluency test. Cognitive decline was defined in two ways: 1) MoCA-defined diagnosis included all PD patients who had a MoCA score < 26; 2) neuropsychological test-defined diagnosis included all PD patients with MoCA-defined diagnosis, who also stated having cognitive decline and had at least two test scores (of HVLT Total Recall, HVLT Recognition Discrimination, Benton Judgement of Line Orientation, Letter Number Sequencing, Semantic Fluency Test and/or Symbol Digit Modalities; irrespective of test domain) greater than 1.5 standard deviation below the age and education-standardised mean score based on published standards in healthy controls, and no functional impairment due to cognitive impairment [31].

### Statistical analysis

Statistical analyses were performed using Statistical Package for the Social Sciences (SPSS), version 22. For all variables, variance homogeneity and Gaussianity were tested using the Kolmogorov–Smirnov test. Continuous variables were expressed as mean ± standard deviation, with between-group comparisons performed by *t* test or Mann–Whitney *U* test for normally or non-normally distributed variables, respectively. Categorical variables were expressed as proportion and compared using a *χ*^2^ test. Predictors of cognitive decline were identified using multivariate backward elimination regression, which included demographic, CSF, plasma and imaging measures. Only the time to occurrence of the first event was used in the backward elimination regression model. In order to determine a characteristic profile for patients most at risk of developing CI, patients were stratified into groups based on the biomarkers that were significant predictors of CI, as defined by the neuropsychological tests. Kaplan–Meier estimates and curves were generated, and comparisons were made using the log-rank (Mantel-Cox) test. Statistical significance was set at *p* < 0.05.

## Results

### Characteristics of PD patients

We studied 262 PD patients who had a mean disease duration of 6.68 ± 6.87 months (Table 1). At the 36-month follow-up, 108/262 (41.2%) PD patients had MoCA-defined CI, of which 40/108 (37.0%) had neuropsychological test-defined CI. Patients who were cognitively impaired as defined by the MoCA were significantly older at baseline and at PD onset and had reduced CSF Aβ42 (*p* < 0.05; Table 1).

### Predictors of cognitive decline

Backward elimination regression analysis for cognitive impairment defined by MoCA did not reveal any predictors of CI (Table 2). Backward elimination regression analysis for cognitive impairment defined by the neuropsychological tests revealed the following predictors, after adjusting for age and gender: CSF Aβ42 (hazard ratio [HR]: 0.996, Wald: 5.035, confidence interval [CI]: 0.992–0.999, *p* = 0.025), CSF total tau ([HR]: 1.023, Wald: 4.680, [CI]: 1.002–1.044, *p* = 0.031) and caudate [^123^I]FP-CIT uptake ([HR]: 0.332, Wald: 4.146, [CI]: 0.115–0.960, *p* = 0.042; Table 2).

**Table 2:** Backward elimination regression analysis for cognitive decline as defined by the MoCA and neuropsychological tests

	HR	95.0% CI for HR		*p* value	Wald
		Lower	Upper		
**MoCA-defined CI**					
CSF Aβ1–42 levels pg/mL	0.998	0.996	1.000	0.092	2.840
CSF total tau pg/mL	1.009	0.995	1.023	0.217	1.522
Caudate [^123^I]FP-CIT	0.945	0.538	1.660	0.843	0.039
Age at screening	1.048	1.024	1.073	< 0.001	15.377
Gender	1.318	0.791	2.195	0.289	1.123
**Neuropsychological test-defined CI**					
CSF Aβ1–42 levels pg/mL	0.996	0.992	0.999	0.025	5.035
CSF total tau pg/mL	1.023	1.002	1.044	0.031	4.680
Caudate [^123^I]FP-CIT	0.332	0.115	0.960	0.042	4.146
Age at screening	1.076	1.028	1.126	0.001	10.084
Gender	1.479	0.626	3.490	0.372	0.797

Patients were then divided into subgroups according to: CSF Aβ42, CSF total tau and caudate [^123^I]FP-CIT uptake. We divided these variables into deciles and identified the deciles with the highest power of predication, providing us with ideal cutoff values for each variable. We found that the best cutoff for CSF Aβ42 was 384.6 pg/mL, 45 pg/mL for CSF total tau and 1.82 for caudate [^123^I]FP-CIT uptake.

**Fig. 1:**
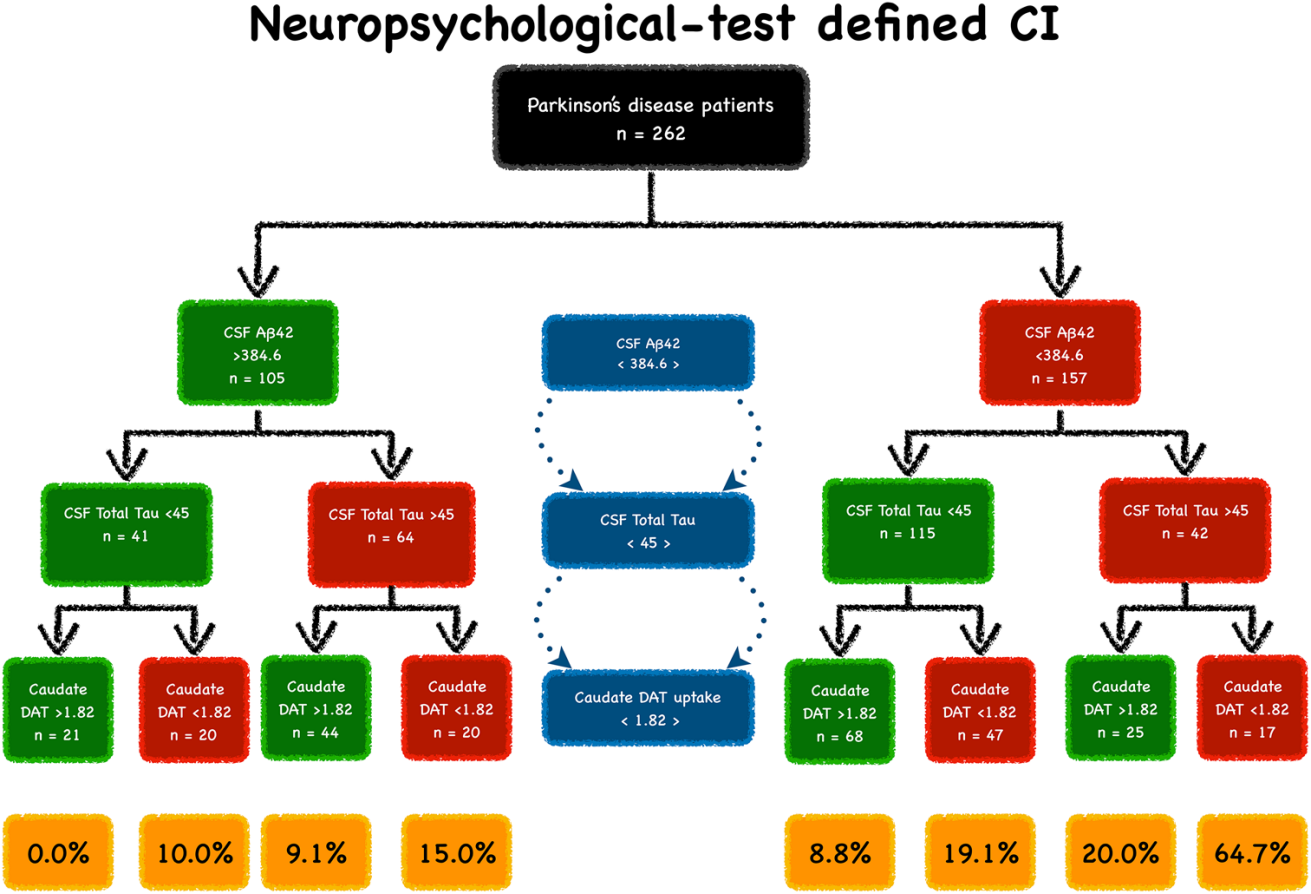
PD sub-phenotypes at varying risk of CI development. PD patients were grouped by CSF Aβ42 (cutoff 384.6 pg/mL), CSF total tau (cutoff 45 pg/mL) and caudate [^123^I]FP-CIT uptake (cutoff 1.82). Green boxes indicate normal variables and red boxes indicate abnormal boxes. The percentages of CI development (yellow boxes) are shown at the end of the study (36-month follow-up)

**Fig. 2:**
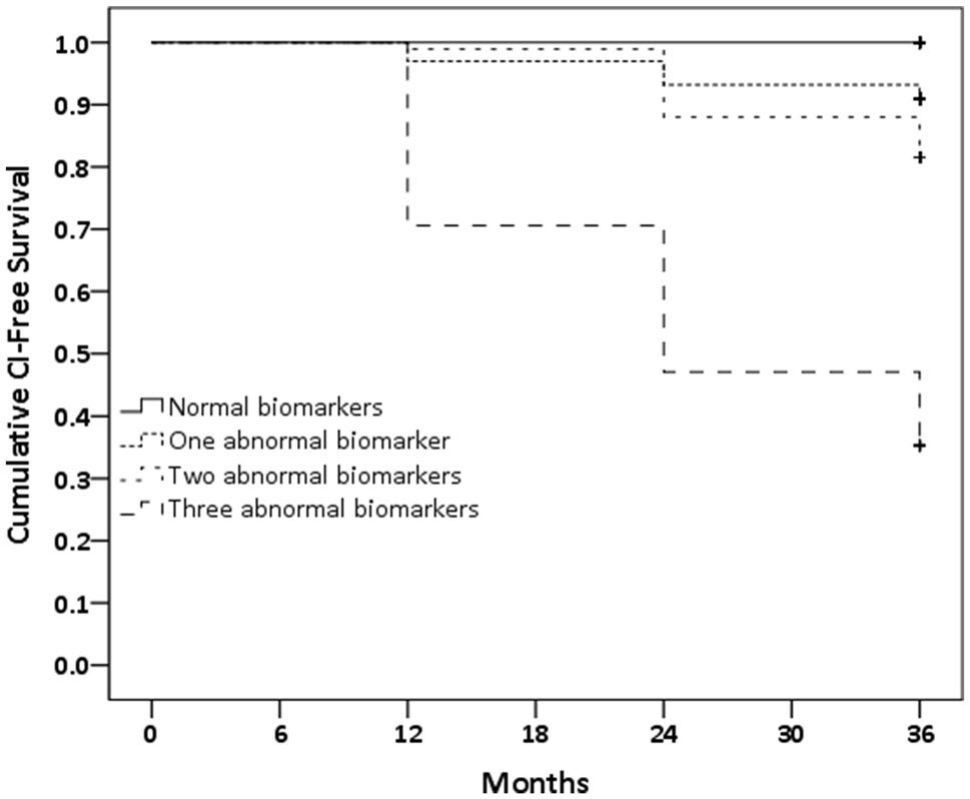
Kaplan–Meier overall survival curves for the development of CI for each subgroup. Log Rank (Mantel–Cox) = 53.03 *p* < 0.0001. *CI* cognitive impairment

PD patients with CSF Aβ42 < 384.6 pg/mL, CSF total tau > 45.0 pg/mL and caudate [^123^I]FP-CIT uptake < 1.82 had a 65% risk of developing CI, as defined by the neuropsychological tests, at a 36-month follow-up (Fig. 1). Patients with normal biomarkers did not develop CI. Patients with only one abnormal biomarker had an 8.8–10.0% risk of developing CI over a period of 36 months. Patients who had a combination of two abnormal markers had 15.0–20.0% risk of CI development over a period of 36 months (Fig. 2).

## Discussion

This pilot study demonstrated that a combined evaluation of biological and neuroimaging markers enables the identification of 65% of drug-naïve PD patients that will develop CI over a 36-month follow-up. CSF Aβ42, CSF total tau and caudate [^123^I]FP-CIT uptake are predictors of CI. According to these measures, PD patients can be categorised into eight subgroups—each with a different conversion rate to CI depending exclusively on how many abnormal measures they have (Fig. 1). Patients with reduced CSF Aβ42, increased CSF total tau and reduced caudate [^123^I]FP-CIT uptake have a 65% conversion to CI, whereas patients who only have one abnormal measure have between 8.8 and 10.0% conversion rate to CI. These findings confirm previous reports of low CSF Aβ42, elevated CSF total tau and reduced dopaminergic integrity being associated with cognitive decline in PD [8, 17, 25, 28].

Although we did not find any predictors for MoCA-defined CI, this may be due to the fact that these particular biomarkers were not sufficient to detect a change in MoCA scores over a 36-month period. Furthermore, neuropsychological test-defined CI was done by a subjective complaint of cognitive decline by the patient at the visit follow-up and *via* a neuropsychological battery, which was selected to identify cognitive deficits typically occurring in PD, independent of motor abilities. This, therefore, suggests that the biomarker profile reflects subtle, accurate cognitive changes in PD, as opposed to a loss of four points on the MoCA.

We propose that the current findings provide a simple characteristic profile of PD patients at risk of CI, which could not only be utilised in clinical settings to identify and correctly monitor PD patients who are most at risk of developing CI, but offer the opportunity to inform treatment decisions and bring personalised medicine into clinical practice. When taken as single markers, CSF Aβ42, CSF total tau and caudate [^123^I]FP-CIT uptake are inadequate to identify PD patients at higher risk of cognitive decline, but taken together, these measures are able to predict cognitive decline in 65% of the cases.

Our findings are in line with the Norwegian ParkWest study, which reported a relationship between reduced CSF Aβ42 levels and cognitive deficits in the memory domain [3], as well as lower CSF Aβ42 levels predicting the development of dementia in PD [4]. Other studies that enrolled more advanced PD patients also concluded that PD patients exhibiting low CSF Aβ42 levels at baseline have an increased susceptibility of developing CI [9]. Previous, smaller longitudinal studies demonstrated that caudate DAT binding can predict cognitive decline [23] and higher CSF total-tau and phospho-tau have an association with recall, naming, recognition and visuoperceptive neuropsychological deficits [8]. Our findings also corroborate with the study conducted by Caspell-Garcia and colleagues [7], who similarly assessed predictive biomarkers of cognitive decline in de novo PD patients from PPMI. Caspell-Garcia et al. [7] reported that reduced striatal DAT availability and low CSF Aβ42 levels predicted cognitive decline, alongside diffuse cortical thinning and two SNPs: the *COMT* val158met and *BDNF* val66met, though increased CSF total tau was not found to be a predictor of cognitive decline. The novelty of our study is that it was designed to identify cutoffs for the predictive biomarkers, which could potentially be employed in the clinical setting. Once validated in other cohorts, these cutoffs provide a clinically-relevant profile that can be employed in clinical trials.

By revealing a characteristic profile of PD patients most at risk of developing CI, it may propel development of mechanism-specific interventions through the identification of suitable subgroups for clinical trials and provision of objective measures of disease suppression. Objective, quantifiable characteristics of pathological processes are particularly crucial for future disease-modifying therapy trials for patients at high risk of cognitive decline. The high attrition rate for drugs in clinical development is predominantly due to the inability of the industry to predict a drug candidate’s performance early, and with a large degree of certainty. Biomarkers hold the potential to substantially accelerate product development and reduce drug development costs by predicting drug efficacy and discriminating between suitable or unsuitable candidates for a given drug. Success with new therapies would rapidly lead to the widespread application of biomarkers in the clinical management of cognitive disorders in PD. Here, we have provided a combination of biomarkers which, taken together, have the ability to predict CI in 65% of early PD cases. This predictive, characteristic profile could tackle the challenges attached to clinical trials by enabling the correct identification of appropriate candidates for trials aiming to prevent or delay cognitive decline in PD.

We show that [^123^I]FP-CIT deficit in the caudate is vital for CI development. Caudate dopaminergic function has been recognised as a fundamental node in supporting cognitive domains. In people exposed to the neurotoxin 1-methyl-4-phenyl-1,2,3,6 tetrahydropyridine (MPTP), which causes a selective dopaminergic lesion, deficits in visuospatial and executive functions were noted [29]. Patients that had a stroke of the caudate showed a deterioration in their intellectual function within 2 years of the event [6]. Previous findings have also illustrated that loss of dopaminergic projections to the caudate correlate with degree of dementia in PD [26], alongside others reporting that a reduction in caudate uptake of [^18^F]DOPA is associated with verbal and visual memory functioning, verbal fluency and, delayed recall [25], highlighting that the functional integrity of presynaptic dopaminergic synthesis is crucial, not only for motor, but also for cognitive function in PD. Although the putamen has inherited the motor functionality classically ascribed to the basal ganglia, studies have demonstrated a role for the putamen in learning and memory [30]. We did not find an association between [^123^I]FP-CIT putamen binding and cognitive impairment, which is in line with findings from [14, 24], who did not find a correlation between putamen uptake of [^18^F]fluorodopa and deficits in neuropsychiatric assessments. This highlights that correct identification of PD patients at risk of CI should include assessment of [^123^I]FP-CIT uptake in the caudate.

Although inclusions containing α-synuclein is the hallmark pathology of PD, PD is now recognised as a more complex clinicopathological entity, with multiple coexisting processes. Neuropathological studies have highlighted the co-occurrence of α-synuclein inclusions and amyloid plaques in neocortical regions in PD patients. Studies have reported that PD patients with dementia have increased β-amyloid using Pittsburgh Compound B PET imaging and a positive correlation between AD pathology and severity of PD with dementia [13].

Aβ42, in particular, is believed to potentiate α-synuclein aggregation and accumulation of Aβ42 and tau is exacerbated by α-synuclein. Furthermore, Aβ42 can contribute to the development of Lewy body disease through promoting α-synuclein aggregation, supporting the role of AD pathology in the pathogenesis of cognitive decline [2]. Reduced CSF Aβ42, however, likely reflects the exacerbated accumulation of Aβ42 in the brain. Preclinical studies have suggested that plaques act as an Aβ42 “sink”, therefore preventing soluble Aβ42 from being transported from the brain to the CSF [10]. Fagan was the first study to investigate this relationship in humans, revealing that low CSF Aβ142 reflect higher levels of Aβ42 deposition in the cortex of AD patients, supporting the hypothesis [11]. No data, however, has been showed in PD.

The microtubule-associated protein tau gene (*MAPT*) has been consistently associated with PD risk, with studies reporting cross-sectional reductions in CSF total tau in PD patients compared to controls [21]. Increased CSF total tau has been suggested to reflect neuronal, preferentially axonal, damage or degeneration, therefore inducing cognitive impairment. Although studies have reported the independent predictive nature of CSF Aβ142 [4], Compta and colleagues reported high levels of CSF tau in PD patients with dementia, compared to non-demented PD patients [8]. Recently, Gomperts and colleagues imaged tau in patients with Lewy body diseases using a highly selective radioligand: [^18^F]AV-1451 [12]. They reported that patients with Lewy body dementia and cognitively impaired PD patients had increased [^18^F]AV-1451 binding in the inferior temporal gyrus and precuneus, which was associated with increased cognitive impairment.

The mechanisms underlying early cognitive impairment in PD have been demonstrated to be multifactorial. In addition to caudate dopaminergic dysfunction and comorbid AD pathology, the underlying pathology of cognitive impairment in PD in also believed to include non-dopaminergic neurotransmitter dysfunction (serotonin, norepinephrine and acetylcholine), cortical/limbic Lewy body pathology and cerebrovascular damage, as well as cortical atrophy and genetic factors. However, PD patient with low caudate [^123^I]FP-CIT uptake, low CSF Aβ142 and elevated CSF total tau may represent a distinct subset of subjects at higher risk of cognitive decline. Lashley and colleagues put forward the notion that subgroups of PD patients could be differentiated based on significant quantitative differences in their cortical Aβ burden, which is in turn associated with α-synuclein load in cerebral cortex [20].

The major limitation of this study is related to the grouping. This method is relatively stringent in terms of inclusion characteristics. Although this is beneficial for controlling the variables hypothesised to be predictive of cognitive decline, it has led to a restricted number of patients within each cohort. Furthermore, given the lack of adjustment for multiple testing, it is important to note that these results are highly exploratory, and therefore require validation from larger studies. It is also imperative to highlight that the cognitive battery was limited, whereby there was a lack of coverage of certain domains such as language abilities, and an uneven coverage of other domains. Although this level of assessment provided a lower diagnostic certainty than a more extensive neuropsychological evaluation, the cognitive tests included are easy to administer and freely available. Furthermore, classification of CI was done according to a predefined criterion that has been employed across several studies exploring CI in PD [7, 31]. Furthermore, although at the time of follow-up, the patients were classified as having developed cognitive impairment, we were not able to account for the possibility of patients reverting back to a cognitively normal condition after 36 months, which may have been due to initiation/ change of treatment or mood fluctuations.

CSF is an accessible source of brain-derived proteins, which reflect molecular changes in the brain and [^123^I]FP-CIT SPECT provides valuable information on brain structure and function in the neuropsychiatric aspects of PD. Reduction of [^123^I]FP-CIT uptake within the putamen is commonly used in clinical practice to assist the diagnosis of PD and here we demonstrated a potential clinical applicability of [^123^I]FP-CIT uptake within the caudate assisting with disease prognosis, specifically cognitive decline. We show that having low CSF Aβ42, elevated CSF total tau, and low [^123^I]FP-CIT caudate binding increase the risk of cognitive decline in PD patients by up to 65%. These measures, taken together, may also reflect a characteristic profile of early PD patients most at risk of developing CI over the course of the disease. However, given the restricted number of patients within each subgroup, further studies with larger cohorts of PD patients are required to confirm and validate these exploratory findings.
